# Aberrant Promoter Methylation of Caveolin-1 Is Associated with Favorable Response to Taxane-Platinum Combination Chemotherapy in Advanced NSCLC

**DOI:** 10.1371/journal.pone.0107124

**Published:** 2014-09-15

**Authors:** Seth A. Brodie, Courtney Lombardo, Ge Li, Jeanne Kowalski, Khanjan Gandhi, Shaojin You, Fadlo R. Khuri, Adam Marcus, Paula M. Vertino, Johann C. Brandes

**Affiliations:** 1 Atlanta VA Medical Center, Atlanta, Georgia, United States of America; 2 Departments of Hematology and Medical Oncology, School of Medicine, Emory University, Atlanta, Georgia, United States of America; 3 Department of Radiation Oncology, School of Medicine, Emory University, Atlanta, Georgia, United States of America; 4 Department of Human Genetics, School of Medicine, Emory University, Atlanta, Georgia, United States of America; 5 Department of Pathology, School of Medicine, Emory University, Atlanta, Georgia, United States of America; 6 Winship Cancer Institute, Emory University, Atlanta, Georgia, United States of America; 7 Department of Biostatistics and Bioinformatics, Rollins School of Public Health, Atlanta, Georgia, United States of America; Virginia Commonwealth University, United States of America

## Abstract

**Purpose:**

Aberrant promoter DNA methylation can serve as a predictive biomarker for improved clinical responses to certain chemotherapeutics. One of the major advantages of methylation biomarkers is the ease of detection and clinical application. In order to identify methylation biomarkers predictive of a response to a taxane-platinum based chemotherapy regimen in advanced NSCLC we performed an unbiased methylation analysis of 1,536 CpG dinucleotides in cancer-associated gene loci and correlated results with clinical outcomes.

**Methods:**

We studied a cohort of 49 patients (median age 62 years) with advanced NSCLC treated at the Atlanta VAMC between 1999 and 2010. Methylation analysis was done on the Illumina GoldenGate Cancer panel 1 methylation microarray platform. Methylation data were correlated with clinical response and adjusted for false discovery rates.

**Results:**

Cav1 methylation emerged as a powerful predictor for achieving disease stabilization following platinum taxane based chemotherapy (p = 1.21E-05, FDR significance  = 0.018176). In Cox regression analysis after multivariate adjustment for age, performance status, gender, histology and the use of bevacizumab, CAV1 methylation was significantly associated with improved overall survival (HR 0.18 (95%CI: 0.03–0.94)). Silencing of CAV1 expression in lung cancer cell lines(A549, EKVX)by shRNA led to alterations in taxane retention.

**Conclusions:**

CAV1 methylation is a predictor of disease stabilization and improved overall survival following chemotherapy with a taxane-platinum combination regimen in advanced NSCLC. CAV1 methylation may predict improved outcomes for other chemotherapeutic agents which are subject to cellular clearance mediated by caveolae.

## Introduction

With the exception of patients whose tumors harbor a targetable driver mutation, response rates following first-line chemotherapy in patients with advanced non-small lung cancer (NSCLC) remain poor[1] [2]. Predictive biomarkers hold the promise to better select patients for specific cytotoxic chemotherapy agents, enabling the physician to choose the most appropriate treatment regimen, thus improving overall response rates and preventing unnecessary toxicity. In modern combination regimens, taxanes are the class of drugs most commonly combined with a platinum backbone. Alternatives include pemetrexed, vinorelbine or gemcitabine. The availability of these active alternatives justifies an effort to identify biomarkers that are predictive of improved response and survival following taxane- or pemetrexed based chemotherapy in NSCLC. Expression of thymidylate synthase has been shown to be a predictor of pemetrexed sensitivity [3].

We have recently identified reduced protein expression of the mitotic checkpoint gene CHFR as powerful predictor of taxane sensitivity in NSCLC [4]. Patients with reduced CHFR expression had a significantly higher likelihood of achieving a clinical benefit and had significantly improved overall survival. Challenges in standardizing and quantifying immunohistochemistry for CHFR, however, are potential limitations of this biomarker.

The detection of aberrant promoter methylation and subsequent epigenetic silencing of genes involved in the cellular response to chemotherapy has been proposed as a qualitative biomarker for chemotherapy response [5]. The major advantage of this approach is that the detection of aberrant methylation by PCR based assays is easier than the detection of a reduction in protein expression and less susceptible to variations in experimental conditions compared with IHC [6]. Well established examples for the role of promoter methylation in the prediction of chemosensitivity are a) MGMT methylation for the prediction of response to alkylating agents such as temozolomide in glioblastoma multiforme [6], [7], b) FANCF methylation as predictor for platinum sensitivity in ovarian cancer [8] and c) CHFR methylation as predictor for taxane sensitivity in gastric [9], cervical [10] and possibly also colon cancer [11].

To identify novel predictive methylation markers for improved outcomes after taxane-based chemotherapy in NSCLC, we performed an unbiased methylation analysis of 1,536 CpG dinucleotides on the Illumina GoldenGate methylation array and correlated results with clinical outcome data amongst NSCLC patients who had received platinum/taxane chemotherapy.

## Materials and Methods

### Study design

The study was approved by the Institutional Review Board of Emory University and the Research and Development Committee of the Atlanta VAMC. Waivers for informed consent requirements were granted due to the retrospective and blinded nature of the clinical data to protected health information (PHI). Patients with stage IV NSCLC who received first-line treatment with a platinum-taxane combination between the years 1999–2010 were initially identified from the local cancer registry at the Atlanta VAMC. We had previously correlated CHFR expression with clinical outcomes in this cohort [4]. Given the different requirements for tissue sections, tumor content and amount of available genomic DNA, not all patients with available tissue blocks qualified for both studies. The registry data were then validated by review of the individual medical records. The following variables were recorded: Age, Sex, Race, chemotherapy regimen, number and type of subsequent therapies, clinical response at first restaging exam, ECOG performance status, tumor histology, date of first diagnosis and overall survival. Patients were further categorized based on the ECOG performance status into good (0 and 1) vs. poor (2 and 3) status. Imaging studies were reviewed individually and response assessment was done by “Response Evaluation Criteria In Solid Tumors (RECIST 1.1)” criteria. Patients who had received at least 2 cycles of therapy and had available paraffin-embedded blocks with sufficient tumor tissue to cut at least 4 sections at 5 uM thickness were eligible.

### Histopathology

Paraffin blocks were cut in sections of 5 uM thickness. One slide was stained with hematoxylin and eosin (H&E) and analyzed by microscopy for tumor type and tumor cell percentage. Only samples with at least 40% viable tumor cell content were used for subsequent analysis. At least 2 additional unstained sections were obtained for DNA extraction.

### DNA extraction and RNA extraction

DNA was extracted from slides 2 and 3 using the E.Z.N.A™ FFPE DNA extraction kit from Omega Biotek (Norcross, GA). RNA was extracted from slides 4 and 5 using the E.Z.N.A™ FFPE RNA extraction kit from Omega Biotek (Norcross, GA). Nucleic acid content was quantified using an Eppendorf Biophotometer Plus (Eppendorf, Hauppauge, NJ) with Hellma Tray Cell (Hellma, Mullheim, Germany).

### Quantitative polymerase chain reaction

mRNA was reversed transcribed using a mix of random hexamer and oligo-dT primers using the Super Script III First strand synthesis kit (LifeTechnologies). qRT-PCR was carried out at a Tm of 55C on a Step One Plus thermocycler (Life Technologies). Primer sequences are available upon request.

### Methylation microarray

High-throughput methylation profiling was done using the Illumina GoldenGate Methylation Cancer Panel I microarray platform. DNA quality control by picogreen, bisulfite-conversion by the EZ DNA methylation kit (Zymo, Irvine, CA) and array hybridization, according to manufacturer's specifications, were performed by Emory Integrated Genomics Core facility.

### Statistical analysis

Data preprocessing and normalization of intensities used the methylumi bioconductor package [12]. In specific, samples were removed that did not pass quality control criteria in terms of an average methylation intensity detection p-value of at least 0.15 or had a low tumor content (below 50%), resulting in 33 patient samples (15 progressive disease (PD), 9 partial response (PR) and 9 stable disease (SD)) for analysis of beta values. A three-way analysis comparing mean beta values among PR versus PD versus SD using an F-statistic based on an ANOVA, applying an FDR corrected p-value of less than 0.05, resulted in the selection of two genes, CAV1 and TEK. Partek Genomics Suite Software (Partek, Inc.) was used for generating heat-maps and clustering of results.

The multivariate survival analysis was conducted by entering sex, race, ECOG performance status, age, race, histology, and Avastin use into a Cox proportional hazard model.

### Cell culture

A549, HOP-62 and EKVX NSCLC cell lines were grown in RPMI media, supplemented with 10% fetal calf serum (Invitrogen). All cell lines were a gift from Dr. Paula Vertino who originally obtained the lines from American Type Culture Collection (ATCC), Manassas, VA. Cell lines were authenticated by STR analysis by Biosynthesis Inc. (Lewisville, TX).

### Constructs and transfection

Small hairpin RNA (shRNA) against Caveolin-1 and non-effective scrambled shRNA in pRFP-C-RS vector were obtained from Origene (Rockville,MD). 1.5 µg of vector were transfected into A549, Hop-62 and EKVX cell lines using Lipofectamine-2000 (Invitrogen). Stably transfected clones were selected and expanded after incubation in Puromycin containing selection media (2.4 µg/mL final). Successful transfection of the clones was determined by fluorescent microscopy and visualization of red fluorescence as well as by subsequent immunoblot for caveolin-1.

### Immunoblotting

Cells were lysed in 1x cell lysis buffer (Cell Signaling), containing Complete protease inhibitor and Phostop (Roche) and 1 mM PMSF. Cells were sonicated briefly and lysates clarified by centrifugation. Following SDS-PAGE and semi-dry transfer the following antibodies were used: Caveolin-1 (1∶1000, Cell Signaling), beta-actin (1∶10000 Sigma), E-cadherin (1∶1000 BD), MDR1 (1∶1000 Cell Signaling), Focal-adhesion kinase (FAK) (1∶1000, Cell Signaling), phospho-Y397-FAK (1∶1000, Cell Signaling).

### Colony Forming Assay

Cell lines indicated were seeded at 1000 cells per well in a 6 well cluster plate. 24 hours post seeding, the cells were treated with docetaxel at 50 nM for 1 hour. The media was exchanged and colonies were allowed to develop for up to three weeks. The cells were fixed with 4% formaldehyde/PBS and stained with crystal violet for imaging and analysis. Image J software was used to quantify the results by measuring the area fraction of each well containing colonies after applying a threshold to the images to eliminate background. Data are reported as fold change over mock treated control.

### Wound healing assay

A549 cells lines bearing either shCAV1 or scrambled control were seeded to 75% confluence in 6 well dishes. 16 hours post seeding, the cell monolayers were scratched with a 20–200 ul pipette tip and imaged under 5X power at the indicated time points. The denuded area was measured using Image J software. Technical triplicates were averaged and values reported as area recovered compared to the zero-hour time point.

### Live Cell Imaging

A549 stable cell lines bearing RFP-expressing shCAV1 or scramble controls were enriched to near 100% purity using a BD-FACS Aria cell sorter and subsequently seeded into Cellview 35 mm glass bottom dishes (Greiner Bio-One). The cells were allowed to attach to the plate overnight. The dish was set on a PE Ultraview spinning disc confocal live cell imaging system. Cells were treated with 2 µM Flutax-1(Santa Cruz Biotechnology) and imaged at 20X in both red and green channels at maximum speed. Three fields of each condition were imaged. After 50 cycles, the media containing Flutax was removed and replaced with Flutax-free media. The samples were imaged for an additional 35–50 cycles. The resulting image sets were analyzed using Cell Profiler software. Analysis of Flutax uptake and turnover, termed Flutax flux, was accomplished by selecting all red positive cells as regions of interest and measuring the change of intensity in the green channel in said regions. This intensity change was measured over 50 cycles (approximately 1.2 cycles per min, 45 minutes total). Reported values are intensities averaged over three fields. Areas under the curves were calculated by trapezoid rule [13].

### Correlation of CAV1 expression and overall survival in independent cohorts

We utilized datasets of 1,715 tumors which had previously been profiled by Affymetrix microarray analysis (www.kmplot.com) [14]. 144 of these tumors also had information on overall survival, clinical stage and on the administration of chemotherapy. CAV1 expression (probe ID: 203065_S_at) was divided by the median into high vs. low expression. Survival analysis by Kaplan-Meier and Cox Proportional Hazard analysis with stage and CAV1 expression as multivariable were performed.

## Results

### Methylation microarray analysis identifies CAV1 methylation as predictor of achieving stable disease after platinum-taxane based combination chemotherapy

**Figure 1:**
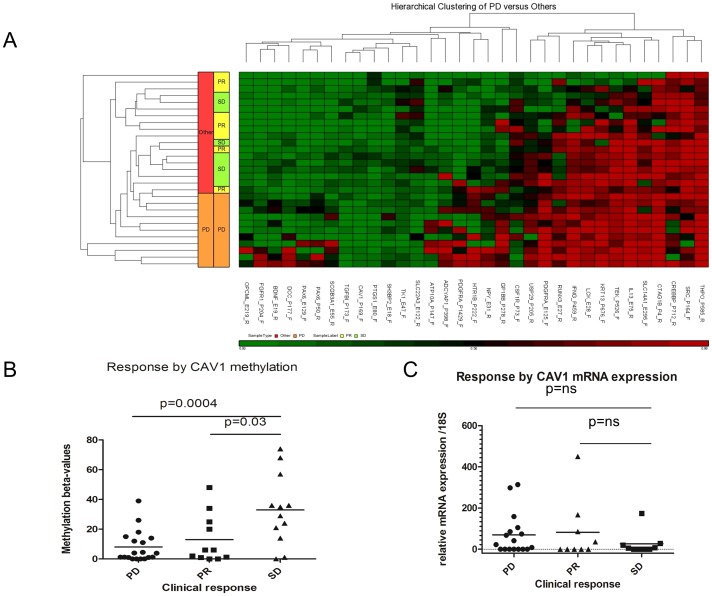
A three way analysis between Progressive Disease (PD), stable disease (SD) and Partial Response (PR) was performed based on an F-statistic based ANOVA with a FDR corrected p-value <0.05. A) Hierarchical clustering of the most prominent changes in relationship at clinical response. B) CAV1 methylation is (by methylation beta-value) is statistically significantly associated with stable disease. C) differences in CAV1 m-RNA expression by qRT-PCR between the three groups of clinical outcomes show a statistically non-significant trend towards decreased expression in patients with SD.

Between the years of 1999 to 2010, a total of 178 patients received platinum plus taxane-based chemotherapy for stage IV NSCLC at the Atlanta VAMC. Of these, 106 had a biopsy confirmation of disease and had received at least 2 cycles of chemotherapy. Paraffin embedded tissue was available for sixty-one of these patients of which forty-six met the inclusion criteria. Thirteen of the samples did not fulfill the quality control criteria for successful hybridization to the array. A total of thirty-three samples were available for final analysis (Table 1). Differential methylation (a comparison of individual probe mean beta values) was then correlated with clinical outcomes (partial response (PR), stable disease (SD) and progressive disease (PD)). Of the 807 genes included on the methylation array, 141 genes correlated to a specific outcome with a p-value <0.05. However after adjustments for false discoveries, only methylation for caveolin-1 (CAV1) (position: −169) and loss of methylation for the TEK receptor tyrosine kinase (position: −526) were statistically significantly correlated with stable disease response (Fig. 1A and 1B). Since loss of methylation is challenging to translate into the development of a clinically useful biomarker, we focused on Cav-1 methylation for subsequent experiments.

**Table 1:** Clinical characteristics.

Sex	Male	49 (100%)
	Female	0(0%)
Age	Median	62 years
	SD	7.6 years
Race	Caucasian	33(67%)
	AA	16(33%)
Histology	SCC	11(22%)
	NOS	15(31%)
	LCC	8 (16%)
	AC	15 (31%)
Chemotherapy	CDDP/TAX	41 (84%)
	CDDP/TAX/Bev	8 (16%)
Overall survival	Median	0.59 years
	SD	0.51 years
Response	PR	11 (32%)
	SD	15 (24%)
	PD	21 (44%)
PS	0	13(28%)
	1	20(44%)
	2	7 (15%)
	3	6 (13%)
CAV1 M	> = 15%	17 (35%)
	<15%	32 (65%)

In order to correlate CAV1 methylation with overall survival in this cohort, we performed univariate and multivariate Cox-regression analyses. In the multivariate analysis and after adjusting for age, performance status, race, histology and the use of bevacizumab, Cav1 methylation was significantly correlated with improved overall survival (hazard ratio (HR) for death: 0.18 (95%CI: 0.03–0.94), p = 0.04) (Table 2), highlighting the potential relevance of Cav1 methylation as clinical biomarker.

**Table 2:** Multivariate analysis.

	HR (95%CI)	p
CAV1 P-169-F	0.18(0.03–0.94)	0.04
PS good vs poor	0.42(0.16–1.13)	0.09
age > = 65 vs <65	0.77 (0.31–1.85)	0.56
Avastin use	0.88(0.33–2.1)	0.78
Race C vs AA	1.88 (0.77–4.95)	0.17
Histo SCC vs non-SCC	1.11(0.40–2.83)	0.83

### Cav1- mRNA expression is reduced in NSCLC with stable disease response, but correlation is weaker than between response and methylation

Promoter methylation generally results in epigenetic silencing of gene transcription [5]. Due to frequent admixtures of stromal or inflammatory cells, methylation analysis frequently correlates better with outcomes following chemotherapy than analysis of mRNA or protein expression [6]. In order to evaluate the correlation between Cav1 methylation and mRNA expression and between mRNA expression and response, we determined Cav1 mRNA levels by qRT-PCR (Fig. 1C). A trend towards reduced Cav1 mRNA levels was observed in patients with stable disease, as would have been expected in specimens with a higher rate of CAV1 promoter methylation, suggesting that CAV methylation may be a more powerful predictive biomarker than CAV1 expression.

### Loss of Cav1 expression induced epithelial mesemchymal transition (EMT)

**Figure 2:**
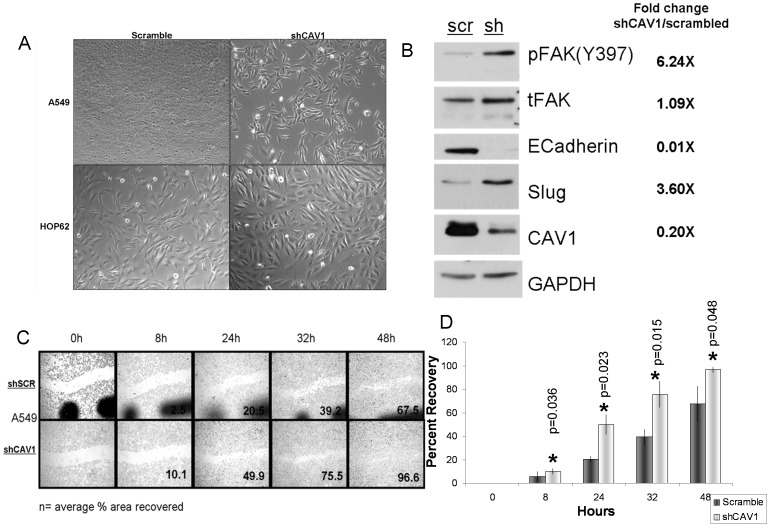
Stable shRNA knockdown induced an epithelial mesenchymal transformation phenotype in A549 cells. A) morphologic changes toward spindle shaped cells after CAV1 shRNA knockdown are observed in A549 cells compared to scrambled shRNA. No such changes are observed in HOP-62 cells which have undergone EMT already. B) CAV1 silencing is associated with reduced expression of E-cadherin and an increase in SLUG expression, both consistent with EMT. Increased FAK-phosphorylation (Y397) signaling serves as a marker for a pro-migratory phenotype C) CAV1 silencing increases cell migration in a wound healing assay D) Quantification of wound healing assays p values (two tailed Student's t) 8 hr p = 0.036, 24 hr p = 0.023, 32 hr p = 0.015, 48 hr p = 0.045.

In order to determine the biologic relevance of a loss of Cav1 expression, we stably transfected A549, HOP-62 and EKVX NSCLC cell lines with a shRNA against CAV1 or non-silencing scrambled controls. In A549 cells, loss of CAV1 was associated with a distinct morphologic change towards spindle cell shape, consistent with EMT (Fig. 2A). At the molecular level, we observed a loss of E-cadherin and an increase of Slugprotein expression consistent with EMT (Fig. 2B). EKVX and HOP-62 are cell lines that display features of EMT at baseline and their phenotype was not altered by CAV1 knockdown. Consistent with the acquisition of an EMT phenotype, shCAV1 transfected A549 cells display increased migration in wound healing assays (Fig. 2C, D). The increased migratory capabilities of CAV1 deficient A549 cells were associated with increased phosphorylation of the focal adhesion kinase (FAK), a well known migration marker [15].

### Loss of CAV1 expression sensitizes lung cancer cell lines to the effects of docetaxel by altering cellular efflux

**Figure 3:**
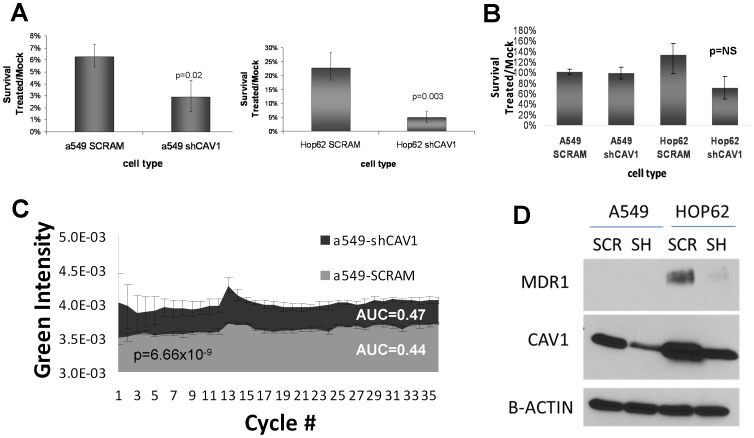
Loss of CAV1 increases taxane sensitivity, by decreasing taxane turnover independent of loss of MDR1. A: CAV1 silencing increases taxane sensitivity in colony formation assays in a549 and HOP62 NSCLC cell lines. Error bars represent standard deviations, p-value <0.05. B: No impact of CAV1 silencing is observed in relationship to cisplatin sensitivity. C: CAV1 silencing leads to increased intracellular taxol concentrations. Live cell microscopy assays were performed in the presence of fluorescently labeled taxol (Flutax). Red- and green fluorescent images were obtained at a frequency of 1.2 images/min. Green fluorescence was analyzed in red-fluorescent cells and plotted over 50 cycles. Statistical analysis was by trapezoid rule (p-value p = 6.66×10^−9^). D: CAV1 silencing is associated with reduced protein expression of MDR-1 in Hop-62 cells but not in A549 cells.

EMT has long been considered to be an important mediator of chemo-resistance in cancer [16], [17], [18], which is in contradiction to the clinical results observed in this study. In order to directly test the role of CAV1 silencing on chemo-sensitivity following exposure to cisplatin and docetaxel, we conducted colony formation experiments on the previously mentioned CAV1 or scrambled shRNA transfected NSCLC lines (A549, HOP62). Interestingly, we observed that CAV1 deficient cell lines were more sensitive to docetaxel than their non-silenced counterparts (Fig. 3A). This is in contrast to treatment with cisplatin which produced comparable cytotoxicity regardless of CAV1 expression (Fig. 3B).

In order to determine if loss of CAV1 expression alters intracellular kinetics of taxane in- and efflux, we performed life-cell imaging after addition of the fluorescently labeled taxol-derivative Flutax-1 (Santa Cruz Biotechnologies). We detected a statistically higher area under the curve (AUC) for Flutax1 in CAV1 deficient cells, suggesting that either increased taxane influx or decreased efflux are likely responsible for the observed increased cytotoxicity in the colony formation assays (Fig. 3C). Multidrug resistance protein MDR1 (also known p-glycoprotein) is an ATPase pump which serves as one of the major cellular detoxifiers. Given its association with caveolae and its known involvement in taxane resistance, expression levels were analyzed and were found to be reduced after CAV1 knockdown in HOP-62 cells (Fig. 3D). However, CAV1 knockdown induced sensitization to taxanes in A549 cells was observed despite a lack of MDR-1 expression, suggesting a mechanism that is at least partially independent from MDR-1 expression.

### Reduced CAV-1 expression predicts improved survival only in NSCLC patients treated with chemotherapy

**Figure 4:**
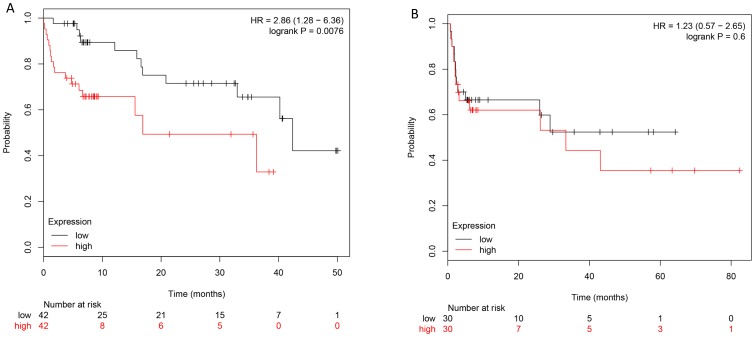
Overall survival was analyzed by CAV1 expression status as determined by Affymetrix gene-expression array in patients who received chemotherapy and those who did not. Out of 1,715 datasets, 144 had clinical information on both stage and overall survival. After adjusting for stage in a multivariate analysis reduced CAV1 expression was associated with improved survival only in patients who received chemotherapy (A) but not in patient who did not (B).

In order to test the hypothesis that CAV1 specifically plays a role in mediating chemoresistance rather than being associated with poor prognosis independently from treatment, we analyzed existing genomically and clinically characterized datasets. After adjusting for stage high CAV1 expression correlated with inferior overall survival in patients who received chemotherapy (HR 2.86; 95% CI 1.28–6.36, p<0.01), but did not predict survival in patients who did not receive chemotherapy (HR 1.23 95%CI 0.57–2.65, p = 0.6) (Fig. 4). These findings support the hypothesis that CAV1 expression is predictive of chemosensitivity and not merely prognostic marker.

## Discussion

This is the first report to show a correlation between promoter methylation of CAV1 with favorable outcomes following combination chemotherapy with paclitaxel and carboplatin in NSCLC. These findings are important because they may help establish CAV1 methylation as clinically relevant biomarker, which could aid in the selection of treatments that lead to a higher likelihood of survival. Our results suggest that CAV1 methylation is a better discriminator for clinical outcomes than mRNA expression. Similar findings have been previously observed for MGMT methylation vs. expression as predictor for response to alkylating agents in glioblastoma [6].

Before CAV1 methylation can be firmly established as predictive marker for taxane sensitivity in lung cancer, three important questions need to be discussed. Is it mechanistically plausible that CAV1 mediates taxane resistance? Is CAV1 silencing specifically associated only with taxane sensitivity or do intracellular pharmacokinetics of other chemotherapeutics converge on the same mechanism? Finally, could CAV1 methylation or expression be a prognostic marker that is associated with favorable prognosis independent of treatment.

CAV1 serves as integral part of caveolae, special lipid rafts which play major functions in cell signaling and endocytosis [19]. In lung cancer both tumor suppressive as well as tumor promoting roles have been described [20]. In small cell lung cancer (SCLC), loss of CAV1expression has been found to promote anchorage independent colony formation. In NSCLC reports about tumor promoting vs. tumor suppressive roles of CAV1 are conflicting and vary among different cell lines used. For example, in H1299 cells CAV1 shRNA knockdown led to a decrease in proliferation [20], while in H460 cells increased metastatic potential and proliferation were observed [21]. In multidrug resistant cell lines, MDR1 co-localized to the low density detergent-insoluble membrane fractions which are characteristic of caveolae, suggesting a possible association between caveolae and multidrug resistance. Our data however prove a direct effect of CAV1 silencing on taxane sensitivity and intracellular uptake or retention in an MDR1 independent fashion, possibly by affecting other multidrug transporters. Support for these findings comes from several reports in the literature of taxane resistant A549 cell lines where CAV1 and MDR1 expression were either discordant or localized to different compartments of the cell membrane [22], [23].

Even though our data show that CAV1 silencing increases taxane- but not platinum sensitivity the possibility exists that intracellular pharmacokinetics of other drugs used in lung cancer therapy such as gemcitabine or etoposide may be dependent on CAV1 mediated mechanisms as well. This is due to the fact that no cellular in-or efflux mechanisms have so far been described that are exclusively specific for taxanes which could at least in part explain the observations that lung cancer patients with high CAV1 expression levels had inferior survival compared to those with low expression when gemcitabine based chemotherapy was given [24].

While we have established CAV1 methylation is a predictive biomarker for taxane based chemotherapy response in lung cancer, a possible prognostic value independent from treatment needs to be considered as well. In NSCLC, CAV1 overexpression has been associated with higher disease stage and inferior survival in patients with adenocarcinoma, but a robust correction of the survival data for stage and treatment was not done in these studies [24], [25], [26]. Our findings and other reports in the literature that CAV1 loss induces EMT and increases proliferation, migration and metastatic potential argue against an inherently better prognosis of CAV1 methylated tumors [21]. Further support comes from our findings that overall survival in untreated patients with lung cancer does not differ by CAV1 expression status.

In summary, we have shown that CAV1 methylation is associated with high rates of stable disease and improved overall survival in patients with advanced NSCLC following chemotherapy with platinum-taxane based regimens. CAV1 methylation could serve as biomarker for taxane sensitivity and could help identify subsets of patients who are more likely to benefit from this cytotoxic chemotherapy.
